# Multilevel Data Encryption Using Thermal‐Treatment Controlled Room Temperature Phosphorescence of Carbon Dot/Polyvinylalcohol Composites

**DOI:** 10.1002/advs.201800795

**Published:** 2018-07-31

**Authors:** Zhen Tian, Di Li, Elena V. Ushakova, Vladimir G. Maslov, Ding Zhou, Pengtao Jing, Dezhen Shen, Songnan Qu, Andrey L. Rogach

**Affiliations:** ^1^ State Key Laboratory of Luminescence and Applications Changchun Institute of Optics Fine Mechanics and Physics Chinese Academy of Sciences Changchun 130033 China; ^2^ University of Chinese Academy of Sciences Beijing 100049 China; ^3^ Center of Information Optical Technologies ITMO University Saint Petersburg 197101 Russia; ^4^ Department of Materials Science and Engineering, and Centre for Functional Photonics (CFP) City University of Hong Kong Kowloon 999077 Hong Kong SAR

**Keywords:** anti‐counterfeiting, carbon dots, data encryption, polyvinylalcohol matrix, room temperature phosphorescence

## Abstract

Thermal‐treatment controlled room temperature phosphorescence is realized by embedding either originally synthesized carbon dots (CDs) or 200 °C thermal‐treated CDs into a polyvinylalcohol (PVA) matrix through post‐synthetic thermal annealing at 200 or 150 °C. The thermal‐treatment controlled phosphorescence is attributed to the transfer of photoexcitation from the excited singlet state to the triplet state through intersystem crossing, followed by radiative transition to the ground state, which is due to decrease of quenchers (oxygen) in the CDs and suppression of the vibrational dissipations through the chemical bonding of CDs in the PVA matrix. Multilevel fluorescence/phosphorescence data encryption is demonstrated based on the thermal‐treatment controlled phosphorescence from CD@PVA composites.

Optical data encryption plays an important role in applications such as document security,^1^ data storage,^2^ and anti‐counterfeiting.^3^ Conventional fluorescent color coding is limited by background interference which demands tedious decryption to extract the coded information.^4^ Thus, it is still a challenge to produce distinguishable coding identities for data encryption, which can be precisely detected and decoded by a simple, rapid, and low‐cost device. Recently, phosphorescent materials have attracted attention as a suitable medium for data encryption, whose long‐living phosphorescence can efficiently eliminate the background interference.^4^, ^5^, ^6^ Conventional phosphorescent materials are usually based on rare‐earth elements; for instance, NaYF_4_:Yb,Tm^4^ and Er^3+^/Yb^3+^ or Tm^3+^/Yb^3+^ co‐doped NaYF_4_ nanoparticles.^6^ Organic phosphorescent materials were also applied as a medium for data encryption.^7^, ^8^ Kwon et al. developed a room temperature phosphorescent system by utilizing strong intermolecular interactions, namely hydrogen and halogen bonds, between the organic phosphor and a polyvinylalcohol (PVA) matrix. These interactions efficiently suppressed vibrational dissipation of charge carriers from the triplet state, which allowed for an increase of the phosphorescence quantum yield up to 24%.^9^ An et al. modulated the structure of organic phosphorescent molecules to tune their color of phosphorescence from green to red for the data encryption.^10^ It is worth noting that rare‐earth elements are expensive and scarce, while organic phosphorescent materials suffer from low thermal and photostability, which constitutes a serious drawback for long time information storage. Therefore, the development of new types of low‐cost, nontoxic, and eco‐friendly phosphorescent materials with high thermal and photostability are an important task for practical data encryption.

Carbon dots (CDs) satisfy the requirements of low toxicity,^11^ biocompatibility,^12^ high photostability,^13^ and low cost,^14^ and offer versatile luminescent properties suitable for a variety of applications.^15^, ^16^, ^17^, ^18^, ^19^, ^20^, ^21^, ^22^ Phosphorescence from CDs in PVA matrix was firstly reported by Zhao's group in 2013.^23^ Since then, several CDs‐based composite systems have been shown to exhibit phosphorescence signal.^24^, ^25^, ^26^, ^27^, ^28^ Dong et al. demonstrated CDs‐based composite powders of potash alum, possessing long phosphorescence lifetimes of 707 ms.^29^ Incorporation of CDs in potash alum resulted in rigidified aromatic carbonyls on the surface of CDs, which prevented nonradiative energy dissipation by rotation or vibration of these groups, and this has been claimed as the origin of the phosphorescence. Li et al. incorporated N‐doped CDs into urea and biuret composite matrices to obtain phosphorescent material with luminescence lifetime of 1.06 s under 280 nm excitation.^30^ Authors claimed that C=N bonds on the surface of CDs create a new energy level and promote the formation of the long‐living triplet excitations, which account for the phosphorescence.

In this work, we demonstrate how the luminescence properties of originally synthesized CDs (denoted as CD‐1) and the same CDs treated at 200 °C (denoted as CD‐2) which are embedded into PVA films (the resulting composites are denoted as CD‐1@PVA and CD‐2@PVA, respectively) can be modulated through the thermal annealing. In the CD‐1@PVA films, enhanced blue fluorescence is observed after 150 °C thermal annealing, while green phosphorescence can only be observed after 200 °C thermal annealing. In comparison, similar phosphorescence can be observed in the CD‐2@PVA film after 150 °C thermal annealing. The thermal‐treatment controlled phosphorescence has been attributed to the transfer of photoexcitation from the excited singlet state to the triplet state through intersystem crossing, followed by radiative transition to the ground state, which is due to decrease of quenchers (oxygen)^23^, ^31^ in the CDs and suppression of the vibrational dissipations through the chemical bonding of CDs in the PVA matrix. Multilevel fluorescence/phosphorescence data encryption has been demonstrated using the CD‐1@PVA and CD‐2@PVA composites.

CD‐1 samples were prepared from citric acid and ammonia water by microwave‐assisted method according to our previous work.^32^ The thermal gravimetric analysis (TGA) data (**Figure** **1**a) illustrates that weight loss of CD‐1 takes place during heating higher than 200 °C, which is due to dehydration and carbonization of CDs sample. The CD‐1 was then heated at 200 °C under nitrogen protection, to produce the sample CD‐2. The morphology of CD‐1 and CD‐2 was characterized using transmission electron microscopy (TEM). According to TEM images (Figure 1b,c) the sizes of CD‐1 and CD‐2 are in a similar range of 1–7 nm. The high resolution TEM (HRTEM) images of CD‐1 and CD‐2 reveal their lattice fringes to be 0.21 and 0.25 nm, which is consistent with the (102) and (100) lattice planes of graphitic carbon,^33^, ^34^, ^35^ respectively. As shown in Figure 1d, the X‐ray photoelectron spectroscopy (XPS) spectra of the CD‐1 and CD‐2 contain three peaks at 284.0, 400.0, and 530.6 eV, which are attributed to C_1s_, N_1s_, and O_1s_, respectively.^36^, ^37^ The oxygen content decreases in CD‐2. The C_1s_ spectra (Figure 1e) show four peaks at 284.5, 285.5, 286.8, and 288.6 eV, which are attributed to C—C/C=C, C—N, C—O, and C=O, respectively.^38^ The calculated C(C—O) content in CD‐2 is lower than in CD‐1. The O_1s_ spectra (Figure 1f) show two peaks at 531.8 and 532.8 eV, which are attributed to the O=C and O—H/C—O—C bands, respectively.^39^ The relative content of O(O—H/C—O—C) in CD‐2 is lower than in CD‐1. These results demonstrate that dehydration and carbonization happen as a result of annealing of CDs at 200 °C, which is also in agreement with TGA data.

**Figure 1:**
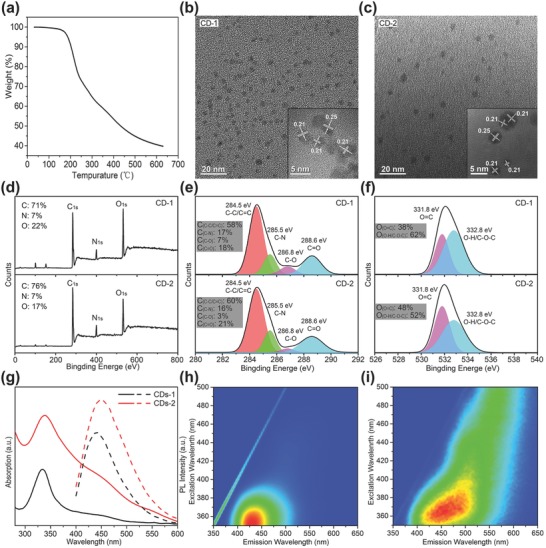
a) TGA curve of CD‐1. TEM images of b) CD‐1 and c) CD‐2 (insets show corresponding HRTEM images). d) XPS survey spectra of CD‐1 and CD‐2. e) XPS C_1s_ spectra of CD‐1 and CD‐2, deconvoluted by four peaks; insets show atomic ratios of C_(C—C/C=C)_, C_(C—N)_, C_(C—O)_, and C_(C=O)_. f) XPS O_1s_ spectra of CD‐1 and CD‐2, deconvoluted by two peaks; insets show atomic ratios of O_(C=O)_ and O_(C—OH/C—O—C)_. g) Absorption (solid) and PL (dashed, taken at 365 nm excitation) spectra of CD‐1 and CD‐2 in water. h,i) Excitation–emission maps of CD‐1 and CD‐2 in water.

Absorption and PL spectra of CD‐1 and CD‐2 in dilute aqueous solutions are shown in Figure 1g. The absorption band of CD‐2 is greatly enhanced and broadened, particularly in the red shoulder. Considering that the absorption intensity is related to the content of conjugated sp^2^ domains in CDs,^40^, ^41^ it can be inferred that the content of conjugated sp^2^ domains were increased in CD‐2. In Figure 1h,i, excitation–emission maps of CD‐1 and CD‐2 in dilute aqueous solutions are presented, respectively. PL band of CD‐2 is redshifted and broadened compared to that of CD‐1. These changes in emission spectra together with enhanced absorption of CD‐2 in the red spectral region can be attributed to the increased conjugated sp^2^ domains. Taking into account XPS analysis, it can be assumed that these increased conjugated sp^2^ domains in CD‐2 are formed mainly by further carbonization at 200 °C with decrease of the C—O and O—H bonds as compared to CD‐1.^41^ We note that neither for CD‐1 nor CD‐2 aqueous solutions no phosphorescence signal was observed at ambient conditions.

Thermal‐treatment controlled luminescence has been studied for CD‐1 and CD‐2 embedded in PVA films. To produce CD‐1@PVA and CD‐2@PVA composites, 0.5 mg of powdered CD‐1 or CD‐2 were dissolved in 2 mL of PVA aqueous solution (10 wt% PVA) and spin‐coated onto quartz plates for further annealing at different temperatures. Absorption spectra of CD‐1@PVA and CD‐2@PVA films annealed at different temperatures are presented in **Figure** **2**a,b. We notice that the absorption enhancement of CD‐1@PVA was much stronger than that of CD‐2@PVA after 180 °C annealing. This can be due to the increased size of conjugated sp^2^ domains in CD‐1 in the PVA matrix caused by thermal‐induced carbonization of the core after 180 °C annealing. At the same time, after annealing at 200 °C, absorbance of both CD‐1@PVA and CD‐2@PVA films were increased, which can be associated with further carbonization of PVA (Figure S2, Supporting Information).

**Figure 2:**
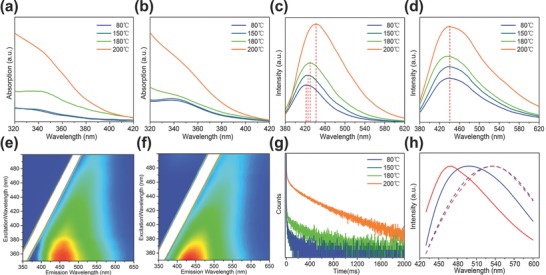
Absorption and c) PL, d) spectra of CD‐1@PVA (a,c) and CD‐2@PVA (b,d) composites at different annealing temperatures (365 nm excitation). Excitation–emission maps of e) CD‐1@PVA annealed at 200 °C and f) CD‐2@PVA annealed at 150 °C. g) Luminescence decay curves collected at 530 nm (405 nm excitation) for the CD‐1@PVA composite annealed at different temperatures. h) Time resolved PL spectra (405 nm excitation) of CD‐1@PVA annealed at 200 °C (red curves) and CD‐2@PVA annealed at 150 °C (blue curves), detected at 1 ns (solid), and 0.2 µs (dashed).

Bare PVA film, used as reference, does not show any emission signals under UV excitation at the annealing temperatures used, as shown in Figure S1 (Supporting Information). On the contrary, CD‐1@PVA and CD‐2@PVA composites show blue emission under UV excitation, indicating the luminescence originates from the embedded CDs. The emission intensity of CD‐1@PVA and CD‐2@PVA gradually increased with the raise of annealing temperature (Figure 2c and d, respectively). According to our previous work,^32^ the surface of CDs contains abundant hydroxyl and carboxyl groups. Chemical bonding through the dehydration process can happen between hydroxyl groups in PVA and hydroxyl or carboxyl groups on the surface of CDs, resulting in elimination of vibrational and rotational motions in the system and, hence, improving their emission. The emission peak of CD‐1@PVA is gradually redshifted from 420 to 440 nm with increase of the annealing temperature. In contrast, the emission peak of CD‐2@PVA remains almost constant (at 440 nm) under annealing (Figure S3, Supporting Information). The redshift of the PL peak of the CD‐1@PVA composite can be attributed to the increased size of sp^2^ domains of these CDs in the PVA matrix, along with increase of the absorbance and luminescence. This is further illustrated by the excitation–emission map of CD‐1@PVA annealed at 200 °C (Figure 2e): PL band moves to green region of the spectrum as compared to CD‐1@PVA film annealed at 80 °C (shown in Figure S4, Supporting Information), which is similar to the excitation–emission map of CD‐2@PVA annealed at 150 °C (Figure 2f).

We have further proceeded to study phosphorescence properties of the composite films. After annealing at 200 °C, green phosphorescence of the CD‐1@PVA can be easily observed by the naked eye (Figure S5, Supporting Information). Transient analysis of the luminescence signal at 530 nm from the CD‐1@PVA composites annealed at different temperatures is presented in Figure 2g. An average luminescence lifetime is sharply increased (reaching more than 100 ms) for the sample annealed at 200 °C. Green phosphorescence of the CD‐2@PVA composite appears already after annealing at 150 °C. To discern fluorescence and phosphorescence signals, time‐resolved PL spectra were collected from CD@PVA composites. From comparison of the spectra presented in Figure 2h, measured with time delay of 1 ns and 0.2 µs, the short‐living spectra slightly differ for CD‐1@PVA annealed at 200 °C and CD‐2@PVA annealed at 150 °C, while their long‐living spectra are similar.

CDs is a complex system of coupled aromatic carbonyls with different groups on their surface, namely hydroxyl, carboxyl, and amino groups.^33^, ^42^ The energy structure of CDs previously discussed in literature usually includes the sp^2^ domain core^38^ and the additional surface states, related to the different surface groups.^39^ Along with the singlet energy levels, such an energy system may accompany triplet states, which exhibit long values of excitation lifetimes from milliseconds to seconds. Radiative transitions at room temperature from triplet states to the ground state are not a common phenomenon for organic molecules,^9^, ^43^ due to the presence of multiple channels of nonradiative energy dissipation, including dissipation by vibrations/rotations of molecules and quenchers in the system. The elimination or at least the reduction of the abovementioned factors may result in occurrence of phosphorescence at room temperature. As already mentioned above, several studies have shown that incorporation of CDs in suitable matrices can be helpful in this respect.^30^, ^31^ The analysis of optical properties of our CD@PVA samples reveals the influence of the thermal annealing on the occurrence of the phosphorescence, as well. For CD‐1@PVA, phosphorescence appears after annealing at 200 °C, while for CD‐2@PVA, already at 150 °C (**Figure** **3**a). Thermal treatment of CD@PVA composites leads to the dehydration, which occurs between hydroxyl groups in PVA and hydroxyl or carboxyl groups on the surface of CDs.^44^, ^45^ As a result, CDs become chemically bonded in the cross‐linked PVA chains, which results in suppressing of vibrations/rotations of the CDs' surface groups.

**Figure 3:**
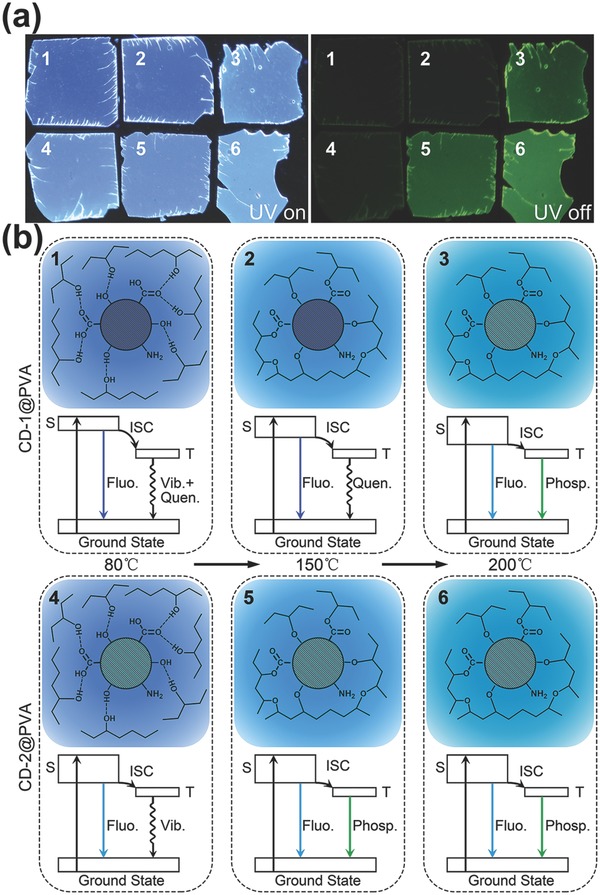
a) Photographs of CD‐1@PVA (samples 1, 2, 3) and CD‐2@PVA (samples 4, 5, 6) composites after annealing at 80 °C (samples 1 and 4), 150 °C (samples 2 and 5), and 200 °C (samples 3 and 6), taken under UV light excitation (left; fluorescence is observed) and after switching UV light off (right; phosphorescence is observed). b) Proposed mechanism of the photophysical processes taking place in CD‐1@PVA and CD‐2@PVA composites annealed at different temperatures: 80 °C (samples 1 and 4), 150 °C (samples 2 and 5), and 200 °C (samples 3 and 6). “*S*,” “ISC,” “*T*,” “*Fluo*.,” “*Phosp*.,” “*Vib*.,” and “*Quen*.” represent “singlet state,” “intersystem crossing,” “triplet state,” “fluorescence,” “phosphorescence,” “vibrational relaxation,” and “quenching,” respectively.

Based on the analysis of the investigated optical properties of CD@PVA composites, a mechanism for the thermal‐induced, enhanced room temperature phosphorescence is proposed and presented in Figure 3b. The energy of the lower electron‐vibrational levels of the CDs can be represented as three levels: ground state, excited singlet state (*S*), and triplet state (*T*). Deactivation of energy from an excited singlet state occurs as a combination of radiative (fluorescence, *Fluo*.) and nonradiative processes. In these processes, an intersystem transition (intersystem crossing, ISC) to the triplet state of the system is involved. From the triplet state, in turn, radiative (phosphorescence, *Phosp*.) and nonradiative transitions are possible. The latter can be associated with two processes: quenching of phosphorescence (*Quen*.) in the presence of certain quenching centers (for example, oxygen in the CDs^23^, ^31^) and energy dissipation associated with the rotational and vibrational motions of the surface and surrounded groups (*Vib*.). Heat treatment of CD samples strongly influences these two processes. As we discussed above, when the initial CDs are annealed at 200 °C, the oxygen amount, which plays the role of a strong phosphorescence quencher in this system,^23^, ^31^ decreases. Therefore, the contribution of nonradiative transitions associated with quenchers for the sample CD‐2@PVA (samples 4–6 in Figure 3) is low, and energy dissipation is dictated mainly by the vibrations/rotations process of the surrounded hydroxyl groups in PVA. In order to get rid of nonradiative dissipation in the vibrations/rotations, it is necessary to chemically bond CDs in PVA network, which was achieved by annealing the CD@PVA samples at temperatures above 150 °C. This is illustrated in Figure 3 for samples 5 and 6. In the sample set 1–3, phosphorescence is observed only for sample 3, which was annealed at 200 °C, which indicates a sequential disappearance of nonradiative transition channels: those associated with the restriction of rotational/vibrational motions of surface groups on the CDs and surrounded hydroxyl groups in PVA, and those associated with decrease of phosphorescence quenchers (oxygen) in the CDs.

The difference in luminescence responses between CD@PVA composites annealed at different temperatures opens up a way for multilevel data encryption. In the following, we propose a concept of the thermal‐treatment controlled multilevel fluorescence/phosphorescence data encryption employing three kinds of composites, multilevel namely blue fluorescent citrazinic acid (CzA@PVA), CD‐1@PVA, and CD‐2@PVA composites. The illustration of the encryption principle is shown in **Figure** **4**. For annealing temperature lower than 80 °C, only blue emissive patterns of indistinguishable information are observed under illumination with UV light, as the encrypted information (in green) written with CD‐1@PVA and CD‐2@PVA does not exhibit phosphorescence signal under UV light. After 150 °C thermal annealing, only the characters written with CD‐2@PVA exhibit green phosphorescence, so that the first level of the encrypted information (characters “*CD*”) written with CD‐2@PVA can be decoded. After 200 °C thermal annealing, both characters written with the CD‐1@PVA and CD‐2@PVA exhibit green phosphorescence, while the intensity of the blue fluorescence from the characters written with CzA@PVA is reduced. Similar decrease in the luminescence signal was observed for CzA@PVA composite annealed at different temperatures, as shown in Figure S6 (Supporting Information). Thus, the second level encrypted information (character “*CDots*”) written with CD‐1@PVA and CD‐2@PVA can be decoded from the low contrast blue fluorescent patterns under UV light and the high contrast green phosphorescent patterns after UV light. It is important that the encrypted information written with CD‐2@PVA can only be decoded after annealing above 150 °C, which means that the first level of information written with CD‐2@PVA (character “*CD*”) cannot be distinguished in the second level encrypted information (character “*CDots*”) after 200 °C annealing. Since it is easy to realize the attachment of PVA‐based composites to glass, plastic, metal, or wood materials, and both the constituting components of CD@PVA composites are biocompatible,^41^ security features based on the above‐illustrated concept can be printed on various media, including merchandise packages, for anti‐counterfeiting and encryption.

**Figure 4:**
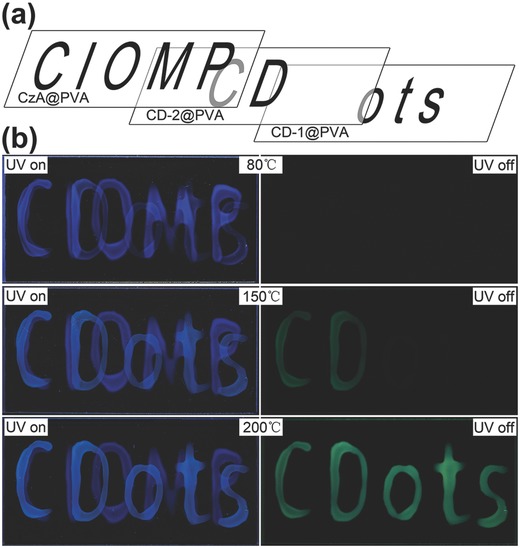
a) Three overlapping patterns written with CzA@PVA (“*CIOMP*”), CD‐2@PVA (“*CD*”), and CD‐1@PVA (“*ots*”) composite. b) Photographs of the combined patterns realized with the mentioned three kinds of composites after annealing at 80, 150, and 200 °C (from top to bottom), under UV light excitation (left) and after switching UV light off (right).

In conclusion, we demonstrated multilevel data encryption using thermal‐treatment controlled room temperature phosphorescence from CD‐1/CD‐2 and PVA composites. Comparing with CD‐1, enhanced green emission of CD‐2 is attributed to the increased size of conjugated sp^2^ domains after 200 °C thermal annealing. In the CD‐1@PVA and CD‐2@PVA films, enhanced blue fluorescence has been observed with increasing of annealing temperature, which has been attributed to chemical bonding of CDs with PVA chains, of the surface groups. In the CD‐1@PVA films, phosphorescence has been observed only after thermal annealing at 200 °C, while phosphorescence in the CD‐2@PVA films has been observed after thermal annealing at 150 °C. The thermal‐treatment controlled phosphorescence has been attributed to the transfer of photoexcitation from the excited singlet state to the triplet state through ISC, followed by radiative transition to the ground state, which is due to decrease of quenchers (oxygen) in the CDs and suppression of the vibrational dissipations through the chemical bonding of CDs in the PVA matrix. A concept of thermal–treatment controlled multilevel fluorescence/phosphorescence data encryption is realized using CzA@PVA, CD‐1@PVA, and CD‐2@PVA composites, which can be utilized for design and development of novel composite materials for anti‐counterfeiting and data encryption.

## Conflict of Interest

The authors declare no conflict of interest.

## Supporting information

Supplementary
