# Supplementary material for: Multilevel Data Encryption Using Thermal‐Treatment Controlled Room Temperature Phosphorescence of Carbon Dot/Polyvinylalcohol Composites
Source: Adv Sci (Weinh). 2018 Jul 31;5(9):1800795. doi: 10.1002/advs.201800795 (PMC6145421; doi:10.1002/advs.201800795)
Supplement: Supplementary file 1 — Supplementary [file ADVS-5-1800795-s001.pdf]

Copyright WILEY-VCH Verlag GmbH & Co. KGaA, 69469 Weinheim, Germany, 2018.

ADVANCED SCIENCE
Open Access

Supporting Information

for *Adv. Sci.*, DOI: 10.1002/advs.201800795

Multilevel Data Encryption Using Thermal-Treatment Controlled Room Temperature Phosphorescence of Carbon Dot/Polyvinylalcohol Composites

Zhen Tian, Di Li, Elena V. Ushakova, Vladimir G. Maslov, Ding Zhou, Pengtao Jing, Dezhen Shen, Songnan Qu* and Andrey L. Rogach

Copyright WILEY-VCH Verlag GmbH & Co. KGaA, 69469 Weinheim, Germany, 2016.

Supporting Information

Multilevel Data Encryption Using Thermal-Treatment Controlled Room Temperature Phosphorescence of Carbon Dot/Polyvinylalcohol Composites

Zhen Tian, Di Li, Elena V. Ushakova, Vladimir G. Maslov, Ding Zhou, Pengtao Jing, Dezhen Shen, Songnan Qu* and Andrey L. Rogach

Supporting Information.

Experimental section and six supplementary figures.

Figure S1: Photographs of bare PVA film and CD-1@PVA composites;

Figure S2: Absorption spectra of bare PVA films;

Figure S3: Emission peak positions and the PL intensity of CD-1@PVA and CD-2@PVA composites;

Figure S4: Excitation-emission map of CD-1@PVA composite;

Figure S5: Illustration of anti-counterfeiting using CD-1@PVA composite;

Figure S6: PL spectra of the CzA@PVA composite.

Experimental Section

Materials: Citric acid (99.5%), ammonia water (25%) and citrazinic acid (97%) were purchased from Aladdin. PVA was purchased from Macklin.

Synthesis of CD-1 and CD-2: Citric acid (3 g) was added to 20 mL of ammonia water (25% ammonia by weight) to form a transparent solution, which was heated in a domestic 650 W microwave oven for 5 min, resulting in the formation of dark-brown liquid. The reaction product was diluted in ethanol (30 mL), and was purified by centrifugation (16,000 r·min-1, 20 min) to remove agglomerated particles. The supernatant was freeze-dried to obtain powdered CD-1. To obtain, as-prepared CD-1 (3 g) were heated at 200°C under nitrogen for 30 min, the product was diluted in ethanol (30 mL), and purified by centrifugation. The supernatant solutions were freeze-dried to obtain powdered CD-2.

Fabrication of CzA@PVA, CD-1@PVA and CD-2@PVA composites: Citrazinic acid (CzA, 0.3 mg), CD-1 (0.5 mg) or CD-2 (0.5 mg) were dissolved in 2 mL of PVA aqueous solution (10 wt% PVA) to prepare CzA@PVA, CD-1@PVA and CD-2@PVA composites, which were spin-coated onto quartz plates and annealed at 80°C, 150°C, 180°C and 200°C for 30 min.

Characterization: UV-visible absorption spectra were taken on a Shimadzu UV-3101PC spectrometer. PL spectra were collected on a Hitachi F-7000 spectrophotometer. PL quantum yields were measured by absolute method in a calibrated integrating sphere in the FLS920 spectrophotometer. Time-resolved PL spectra were measured on a Life Spec-II lifetime spectrophotometer (Edinburgh Instruments).TEM was performed on a FEI Tecnai-G2-F20 instrument operated at 200 kV. AFM was performed on a SA400HV instrument with a Seiko SPI3800N controller. Thermogravimetric analysis (TGA) was performed on an American TA Q500 analyzer under nitrogen atmosphere, with the flow rate of 100 mL•min^{-1}. XPS analysis has been done on an ESCALAB MK II spectrometer using Mg as the excitation source. Fluorescence microscopy images were obtained on a C2+ confocal microscope system (Nikon), with emission spectra collected on an Ocean Optics Maya 2000 Pro Fiber and Fu Xiang NOVA-EX Fiber Optic spectrometers.

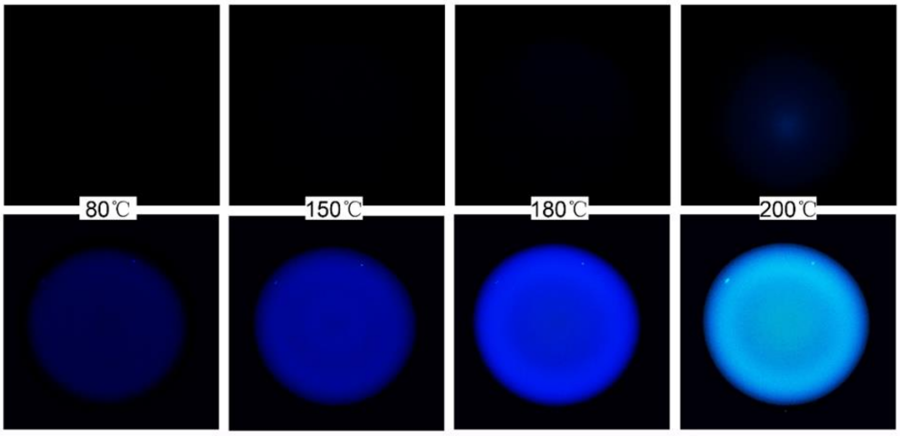

Figure S1. Photographs taken under UV light of bare PVA film (top) and CD-1@PVA

composites (bottom) at different annealing temperatures, as indicated on frames.

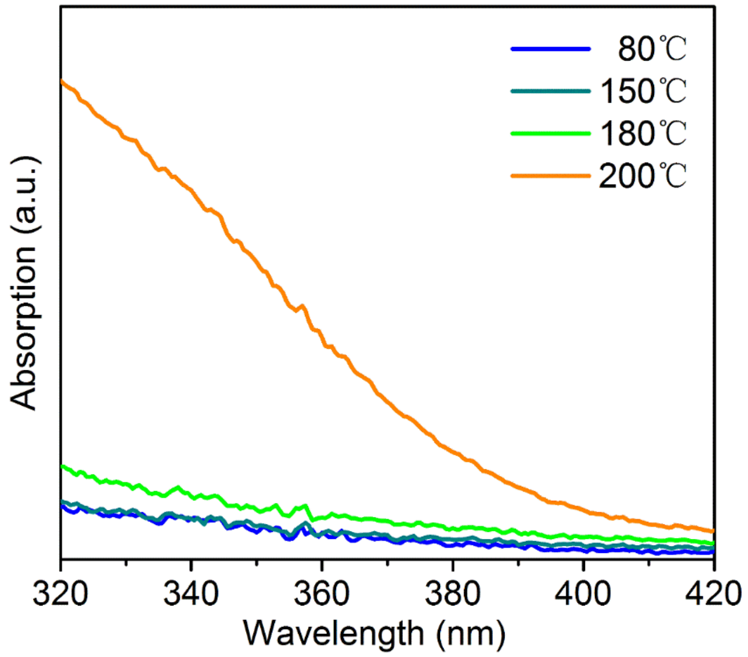

Figure S2. Absorption spectra of bare PVA films at different annealing temperatures, as indicated on frames.

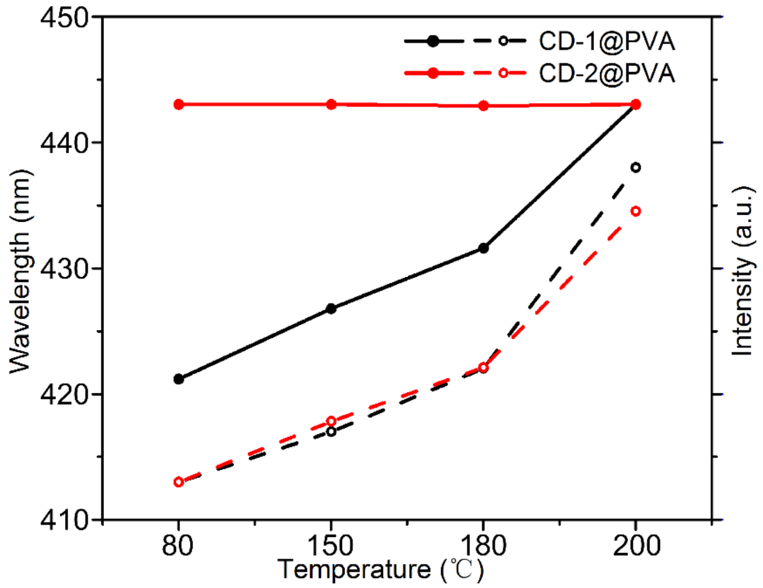

Figure S3. Changes of emission peak positions (solid connecting curves) and the PL intensity (dashed connecting curves) of CD-1@PVA (black symbols) and CD-2@PVA (red symbols) composites as a function of the annealing temperature, under 365 nm excitation.

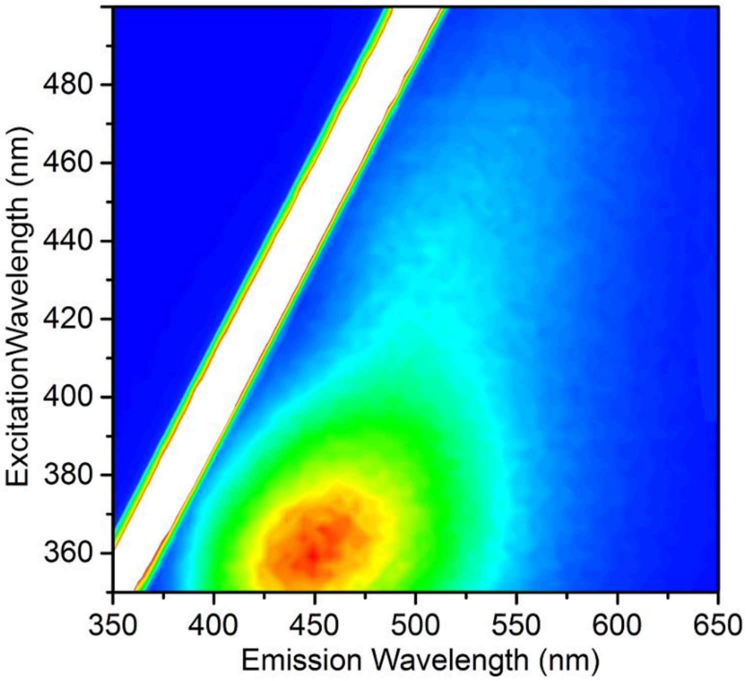

Figure S4. Excitation-emission map of CD-1@PVA composite annealed at 80°C.

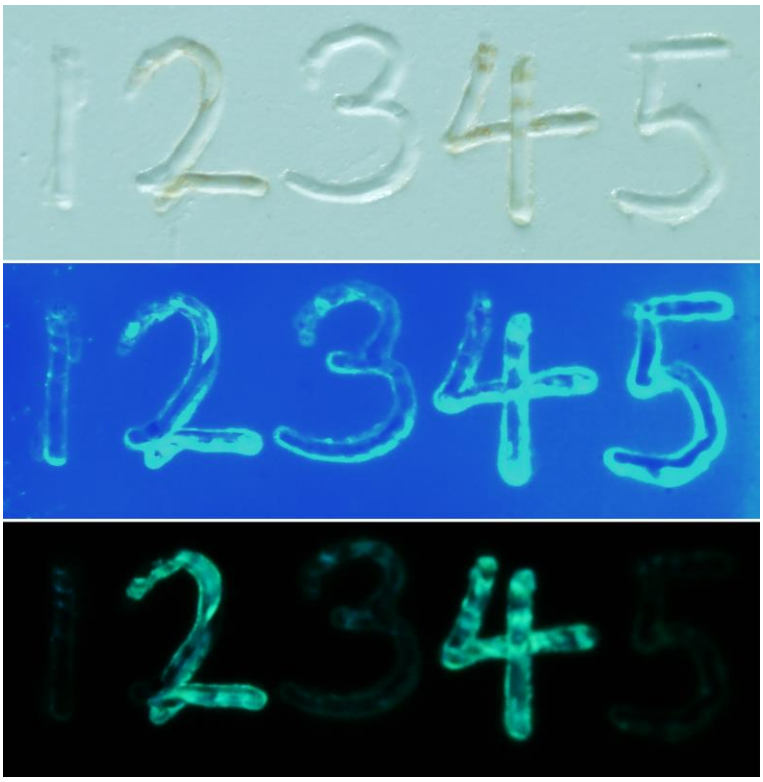

Figure S5. Illustration of anti-counterfeiting using CD-1@PVA composite. CD-1@PVA was coated onto silica gel plate and patterned by hand-writing with electrothermal pen (upper row) at two different temperatures, namely 150°C (characters "1, 3, 5") and 200°C (characters "2, 4"). Under UV light (middle row), all written characters show blue fluorescence. After turning the UV light off (bottom row), only characters written at 200°C show green phosphorescence, while other patterns written at 150°C didn't exhibit room temperature phosphorescence.

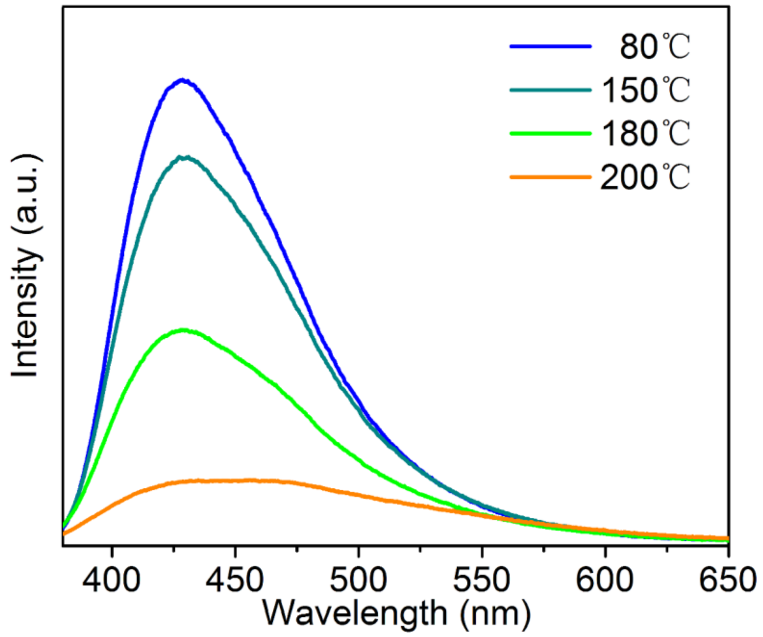

Figure S6. Room temperature PL spectra (365 nm excitation) of the CzA@PVA composite annealed at different temperatures.

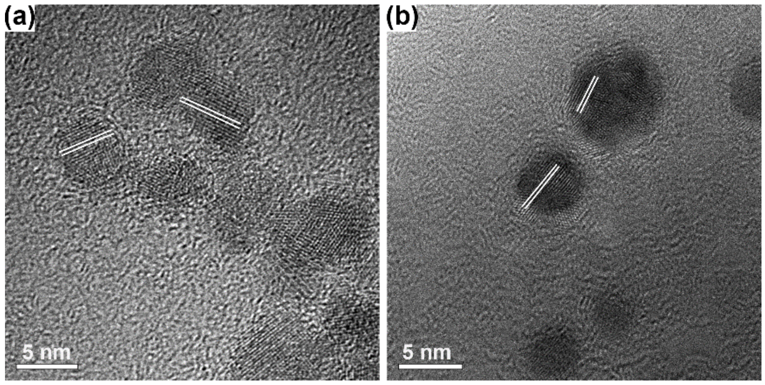

Figure S7. HRTEM images of (a) CD-1 and (b) CD-2
